# Birth weight prediction models for the different gestational age stages in a Chinese population

**DOI:** 10.1038/s41598-019-47056-0

**Published:** 2019-07-25

**Authors:** Chunhui Li, Yang Peng, Bin Zhang, Weiying Ji, Li Li, Jianhua Gong, Wei Xia, Yuanyuan Li, Shuna Jin, Ranran Song, Youjie Wang, Shunqing Xu

**Affiliations:** 1 Key Laboratory of Environment and Health, Ministry of Education & Ministry of Environmental Protection, State Key Laboratory of Environmental Health, School of Public Health, Tongji Medical College, Huazhong University of Science and Technology, Wuhan, Hubei People’s Republic of China; 2 Women and Children Medical and Healthcare Center of Wuhan, Wuhan, Hubei People’s Republic of China; 3 Luohu Maternity and Child Health Care Hospital of Shenzhen, Shenzhen, Guangdong People’s Republic of China; 4 Department of Maternal and Child Health, School of Public Health, Tongji Medical College, Huazhong University of Science and Technology, Wuhan, Hubei People’s Republic of China

**Keywords:** Health care, Statistics

## Abstract

The study aims to develop new birth weight prediction models for different gestational age stages using 2-dimensional (2D) ultrasound measurements in a Chinese population. 2D ultrasound was examined in pregnant women with normal singleton within 3 days prior to delivery (28–42 weeks’ gestation). A total of 19,310 fetuses were included in the study and randomly split into the training group and the validation group. Gestational age was divided into five stages: 28–30, 31–33, 34–36, 37–39 and 40–42 weeks. Multiple linear regression (MLR), fractional polynomial regression (FPR) and volume-based model (VM) were used to develop birth weight prediction model. New staged prediction models (VM for 28–36 weeks, MLR for 37–39 weeks, and FPR for 40–42 weeks) provided lower systematic errors and random errors than previously published models for each gestational age stage in the training group. The similar results were observed in the validation group. Compared to the previously published models, new staged models had the lowest aggregate systematic error (0.31%) and at least a 19.35% decrease; at least a 4.67% decrease for the root-mean-square error (RMSE). The prediction rates within 5% and 10% of birth weight for new staged models were higher than those for previously published models, which were 54.47% and 85.10%, respectively. New staged birth weight prediction models could improve the accuracy of birth weight estimation for different gestational age stages in a Chinese population.

## Introduction

Estimated fetal weight (EFW) is a clinically important indicator to manage perinatal risk and affect the timing and route of delivery^1–4^. Meanwhile, the estimation of fetal weight is helpful to identify small for gestational age (SGA), large for gestational age (LGA), or macrosomia^5–7^. Therefore, a reliable fetal weight estimation is of value for clinicians. Numerous EFW models^8–13^ were developed using ultrasound measurements, such as head circumference (HC), abdominal circumference (AC), biparietal diameter (BPD) and femur length (FL). Most of these models were established based on ultrasound measurements of Caucasians population. However, the NICHD Fetal Growth Studies showed that the fetal weight differed significantly with race after 20 weeks^14^.

Although some researchers developed the EFW models using 2D and 3-dimensional (3D) ultrasound measurements in a Chinese population which were confirmed better than those models based on Caucasians population^15–17^, 2D ultrasound is still widely used in the clinical practice for sonographer compared to 3D ultrasound. Thus, it is necessary to develop more accurate birth weight prediction models based on 2D ultrasound measurements.

In addition, the common feature of these published EFW models is that fetal weight was estimated by single model for the whole gestational age. According to the Intergrowth-21^st^ study^18^, the fetal growth velocity is different depending on gestational age. Moreover, Sotiriadis *et al*.^19^ found that the divergence between birth weight and the EFW based on the Hadlock’s formula is greater for earlier gestational ages, and the same finding was confirmed in a similar study using Norwegian birth registry^20^. It is therefore that the single EFW model may not accurately estimate the fetal weight for different gestational age stages.

We postulate that developing different prediction models for each gestational age stage may improve the accuracy of birth weight prediction in a Chinese population. In this study, we established the staged birth weight prediction models from 28 to 42 weeks using 2D ultrasound measurements in a larger Chinese population, and validated its prediction performance in 28–42 weeks.

## Results

A total of 20,619 women who had an ultrasound scan within 3 days prior to delivery were included, of which 11 women had a stillbirth, 42 women delivered before 28 weeks or after 42 weeks, and 1256 women lacked complete ultrasound measurements (HC, BPD, AC or FL). Ultimately, 19,310 women were analyzed in the study. The clinical and demographic characteristics of women and newborns were shown in Table 1. The median and interquartile range (IQR) of maternal age was 28 and 4 years, respectively. The prevalence rate of gestational diabetes mellitus (GDM) and preeclampsia was 3.66% and 1.95%, respectively. Birth weight had a median of 3300 g and IQR of 560 g. Of 19,310 newborns, 1827 (9.46%) were small for gestational age (SGA, <10th weight percentile for local population^21^), 2790 (14.45%) were large for gestational age (LGA, >90^th^ weight percentile), and 1073 (5.56%) were macrosomia (≥4,000 g). The median gestational age at delivery was 39.43 weeks (ranged from 28 to 42 weeks). Table 1 showed that 987 (5.11%) babies were born preterm (<37 weeks), 194 (1.00%) babies were born term with low birth weight (<2,500 g and ≥37 weeks), and 1,072 (5.55%) macrosomia were born term (≥4,000 g and ≥37 weeks). The mean of ultrasound-to-delivery intervals was 1.23 days (range, 0–3).

**Table 1:** Clinical and demographic characteristics of pregnant women and newborns in the study who had an ultrasound scan within 3 days prior to delivery.

Characteristics	N (%) or Median (IQR)
All	19,310
**Maternal characteristics**	
Age (years)	28 (4)
Weight (kg)^a^	54 (10)
Height (cm)^b^	160 (6)
**Gestational age at birth (weeks)**	
28–30	36 (0.19)
31–33	154 (0.80)
34–36	797 (4.13)
37–39	11,730 (60.75)
40–42	6,593 (34.14)
**Parity** ^**c**^	
1	15559 (80.86)
≥2	3683 (19.15)
**Gestational Diabetes Mellitus**	
Yes	706 (3.66)
**Preeclampsia**	
Yes	377 (1.95)
**Ultrasound to delivery interval (days)**	
0	4,142 (21.45)
1	8,941 (46.30)
2	3,953 (20.47)
3	2,274 (11.78)
**Newborns’ characteristic**	
**Birth weight (g)**	
<1,500	38 (0.20)
1,500–1,999	134 (0.69)
2,000–2,499	518 (2.68)
2,500–2,999	3,512 (18.19)
3,000–3,499	8,886 (46.02)
3,500–3,999	5,149 (26.66)
≥4,000	1,073 (5.56)

Continuous variables are presented as median (IQR: interquartile range); categorical values are N (%); ^a^2606 missing; ^b^5242 missing; ^c^68 missing.

### Demographic information for study population

Of 19,310 subjects, 17,377 cases were randomly assigned to the training group and 1,933 to the validation group. Comparisons of demographic characteristics (maternal age, maternal weight, maternal height, parity, gestational age, ultrasound-to-delivery intervals, newborn’s gender, and birth weight) between the training group and the validation group were shown in Table S1. There were no significant differences for all demographic characteristics in two groups.

### Development of new staged birth weight prediction models

**Figure 1:**
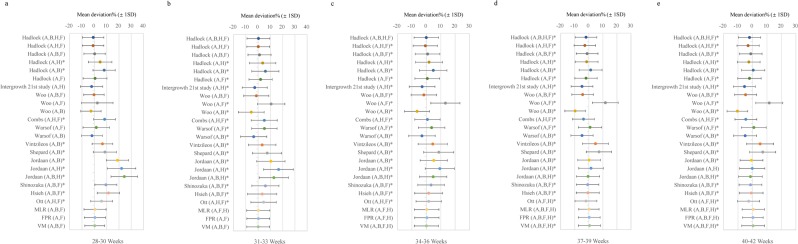
The systematic errors and random errors for established staged birth weight prediction models and previously published models for different gestational age stages in the training group. (**a**–**e**) Represent the systematic errors (±random errors) for 29–30, 31–33, 34–36, 37–39 and 40–42 weeks, respectively. ^*^Indicates that significantly different from zero, *p* < 0.05; A: abdominal circumference (AC); B: biparietal diameter (BPD); H: head circumference (HC); F: femur length (FL); MLR: multiple linear regression; FPR: fractional polynomial regression; VM: volume-based model.

MLR, FPR and VM were used to establish birth weight prediction models for five gestational age stages (Table 2). To compare the performance of new staged birth weight prediction models, 21 previously published formulas were selected (Table S2). Figure 1 showed the comparisons of all birth weight prediction models for five gestational age stages. For first three gestational age stages (28–30, 31–33, 34–36 weeks), the new VM model presented the lowest systematic errors (0.3%, 0.08% and 0.03%, respectively), which were not significantly different from zero (*p* = 0.832; 0.923; 0.918). Compared to the MLR and the FPR models, at least a 67.39%, 1.21 times and 7 times decrease in the systematic errors and a 6.97%, 0.26%, 0.36% decrease in the random errors for the VM model were found in 28–30, 31–33, and 34–36 weeks, respectively. The systematic errors of previously published EFW models were higher than new VM models for 28–30, 31–33 and 34–36 weeks except for Haddock (A,B,F) formula (systematic error: 0.23%) (Fig. 1a–c). For last two gestational age stages (37–39 and 40–42 weeks), the lowest systematic errors (0.09% and 0.29%, respectively) were found in the MLR model for 37–39 weeks and the FPR model for 40–42 weeks, which were significantly lower from those in other two new models and 21 previously published models with *p*-value all <0.05 (Fig. 1d,e). In addition, the random errors in the VM model for first three gestational age stages, the MLR model for 37–39 weeks and the FPR model for 40–42 weeks were the smallest among all the models. Therefore, the VM, MLR and FPR model were used as new staged birth weight prediction models to validate the prediction performance for 28–36, 37–39 and 40–42 weeks, respectively.

**Table 2:** New staged birth weight prediction models for the training group in different gestational age stages.

Gestational age (weeks)	Model	Birth weight prediction models
28–30	MLR	log_10_EFW = 2.42076 + 0.05567 × FL + 0.00221 × BPD × AC
	FPR	log_10_EFW = 2.60972 + 0.38613 × (AC × FL/100)
	VM	EFW = 1.24732 × BPD^3^ + 0.24897 × FL × AC^2^
31–33	MLR	log_10_EFW = 2.62143 + 0.00020423 × HC × AC + 0.00282 × AC × FL
	FPR	log_10_EFW = 2.92746 + 0.652 × log(AC × FL/100)
	VM	EFW = 0.47766 × BPD^3^ + 0.34049 × FL × AC^2^
34–36	MLR	log_10_EFW = 2.75863 + 0.00024933 × HC × AC + 0.00194 × FL × AC
	FPR	log_10_EFW = 0.95627 + 0.5932 × (AC/10)−1.57276 × (AC × HC/1000) + 0.56091 × (HC/10) + 0.18358 × (FL × AC/100)
	VM	EFW = 0.04608 × BPD × HC^2^ + 0.33326 × FL × AC^2^
37–39	MLR	log_10_EFW = 0.50953 + 0.07197 × AC + 0.32308 × FL + 0.00063556 × BPD × AC + 0.00013695 × HC × AC-0.00864 × FL × AC
	FPR	log_10_EFW = 5.686343 − 0.016616 × (AC × FL/100)^3^ − 0.575416 × (AC/100)^−2^ − 0.311367 × (AC/100)^−2^ × log(AC/100) + 0.393768 × (FL × HC/100) − 2.978696 × (HC/100) + 0.065442 × (BPD × HC/100)
	VM	EFW = 0.08619 × BPD × HC^2^ + 0.29568 × FL × AC^2^
40–42	MLR	log_10_EFW = −0.43878 + 0.05966 × HC + 0.07596 × AC + 0.29784 × FL-0.00266 × BPD × HC + 0.00314 × BPD × AC-0.00081197 × HC × AC-0.00815 × FL × AC
	FPR	log_10_EFW = 5.807651 − 0.01166 × (AC × FL/100)^3^ − 0.567751 × (HC/100)^−2^ − 0.287528 × (HC/100)^−2^ × log(HC/100)−0.546421 × (BPD × HC/100) + 3.73769 × (AC × BPD/1000) + 1.003845 × (FL × BPD/100)
	VM	EFW = 0.11314 × BPD × HC^2^ + 0.26401 × FL × AC^2^

Weight is expressed in g; BPD, HC, AC and FL are expressed in cm; MLR: multiple linear regression; FPR: fractional polynomial regression; VM: volume-based model; EFW: estimated fetal weight; HC: head circumference; AC: abdominal circumference; BPD: biparietal diameter; FL: femur length.

### Validation of the different prediction models

**Figure 2:**
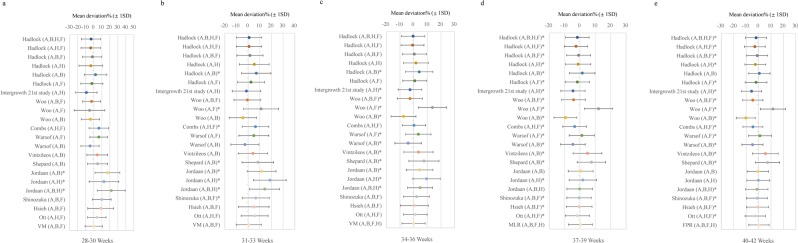
The systematic errors and random errors for new staged birth weight prediction models and previously published models for different gestational age stages in the validation group. (**a**–**e**) Represent the systematic errors (±random errors) for 29–30, 31–33, 34–36, 37–39 and 40–42 weeks, respectively. New staged birth weight prediction models: VM model for 28–30, 31–33 and 36 weeks, MLR model for 37–39 weeks, FPR model for 40–42 weeks. *Indicates that significantly different from zero, *p* < 0.05; A: abdominal circumference (AC); B: biparietal diameter (BPD); H: head circumference (HC); F: femur length (FL); MLR: multiple linear regression; FPR: fractional polynomial regression; VM: volume-based model.

Figure 2 displayed the systematic errors and random errors of different prediction models. In the first three gestational age stages, VM model was selected to compare the prediction performance with 21 previously published models, and in the last two stages, MLR model and FPR model were used, respectively. The lowest systematic errors (−0.08%, 0.51%, −0.09%, 0.36%, and 0.29%) were derived from new staged models for five gestational age stages, and were not significantly different from zero (*p* = 0.989; 0.853; 0.922; 0.116; 0.367) (Fig. 2). In 28–30 weeks, the absolute values of systematic errors for previously published models ranged from 1.98% to 21.81%, which were at least 23.75 times higher than new model (Fig. 2a). In 31–33 weeks, the absolute values of systematic errors of 21 published models (ranged from 0.69% to 18.69%) had a 35.29% increase compared to new model at least (Fig. 2b). Similarly, a 1.33 times decrease in the new model was observed compared to Combs (A,H,F) model with the second lowest systematic error in 34–36 weeks (Fig. 2c). For 37–39 and 40–42 weeks, new models had significantly lower systematic errors than 21 published models (*p*-value both <0.001) (Fig. 2d,e). The random errors of new staged models were close to published models; however, a slight decrease was observed in new staged models for each gestational age stage.

The aggregate systematic errors, random errors, RMSE and prediction rates within 1%, 5% and 10% of birth weight were calculated for each model in the validation group (Table 3). New staged models had the lowest systematic error (0.31%) compared with published models for all fetuses in the validation group. The systematic errors for published models were significantly different from zero with *p*-value all <0.001. The random errors for new staged models were equal or smaller than those for published models, while, RMSE values for new models were lower than those for published models. Hadlock (A,B,H,F) model and new staged models had the same prediction rate within 1% of birth weight (10.86%), which were better than other published models. The prediction rates within 5% and 10% of birth weight for new models were higher than those for other published models, which were 54.47% and 85.10%, respectively.

**Table 3:** Comparisons of staged birth weight prediction models with previously published models for all newborns in the validation group.

Model	Systematic error (%)	Random error (%)	RMSE	Prediction within (%)		
				1%	5%	10%
Hadlock (A,B,H,F)	−1.74*	7.99	282.13	10.86	47.59	78.43
Hadlock (A,H,F)	−2.61*	7.97	292.77	9.26	45.58	76.93
Hadlock (A,B,F)	−0.92*	8.13	278.69	10.24	48.42	78.32
Hadlock (A,H)	−1.54*	8.46	292.72	9.83	46.20	75.79
Hadlock (A,B)	1.35*	8.55	280.58	10.76	46.46	76.10
Hadlock (A,F)	−1.68*	8.34	292.07	9.88	44.90	76.93
Intergrowth 21^st^ study (A,H)	−4.83*	8.12	330.47	7.76	39.47	70.93
Woo (A,B,F)	−4.26*	8.00	319.20	9.00	41.13	72.79
Woo (A,F)	11.93*	9.68	480.18	3.36	19.56	42.83
Woo (A,B)	−9.64*	7.70	437.66	4.45	21.31	48.99
Combs (A,H,C)	−3.60*	8.03	312.09	8.07	43.61	74.13
Warsof (A,F)^a^	1.29*	8.72	290.22	9.42	45.58	76.82
Warsof (A,B)^b^	−4.70*	8.33	333.15	6.73	38.59	69.99
Vintzileos (A,B)	5.02*	9.79	357.17	7.29	36.63	67.20
Shepard (A,B)^b^	7.38*	9.51	378.21	6.47	31.35	60.53
Jordaan (A,B)^b^	0.37	8.55	280.39	10.86	46.67	77.19
Jordaan (A,H)	1.64*	9.44	304.59	8.64	42.47	73.98
Jordaan (A,B,H)	−0.47*	8.87	292.68	9.67	45.99	75.22
Shinozuka (A,B,F)	−0.63*	8.31	281.17	10.45	47.90	77.44
Hsieh (A,B,F)	−0.48*	8.70	289.84	11.02	45.94	75.32
Ott (A,H,F)^b^	−1.84*	8.09	287.09	10.19	47.08	77.86
New staged models	0.31	7.97	266.25	10.86	54.47	85.10

New staged models: VM model for 28–30, 31–33 and 34–36 weeks, MLR model for 37–39 weeks, FPR model for 40–42 weeks; RMSE: root mean square error; *indicates significantly different from zero (p < 0.001); A: abdominal circumference (AC); B: biparietal diameter (BPD); H: head circumference (HC); F: femur length (FL) expressed in cm; ^a^FL expressed in mm; ^b^EFW expressed in kg.

The accuracy parameters (sensitivity, specificity, PPV, NPV, +LR, −LR, and overall accuracy) for each birth weight prediction model for detection of SGA, LGA and macrosomia at birth were shown in Tables 4–6, respectively. For prediction of SGA, LGA and macrosomia, a considerable variation in models’ sensitivity, specificity, PPV, +LR, and −LR were observed; while, a minor variation in models’ NPV and overall accuracy were found. Compared to previously published models, new staged models performed better accuracy for detection of SGA, LGA and macrosomia in Chinese population.

**Table 4:** Accuracy of staged birth weight prediction models for detection of SGA at birth.

Model	Sensitivity (%)	Specificity (%)	PPV (%)	NPV (%)	+LR	−NLR	Overall accuracy (%)
Hadlock (A,B,H,F)	45.90	95.31	50.60	94.4	9.80	0.57	90.64
Hadlock (A,H,F)	51.91	94.51	49.74	94.95	9.46	0.51	90.48
Hadlock (A,B,F)	44.26	96.23	55.10	94.29	11.74	0.58	91.31
Hadlock (A,H)	49.73	95.08	51.41	94.76	10.12	0.53	90.79
Hadlock (A,B)	32.24	97.31	55.66	93.21	12.00	0.70	91.15
Hadlock (A,F)	47.54	95.54	52.73	94.57	10.67	0.55	91.00
Intergrowth 21^st^ study (A,H)	65.57	88.11	36.59	96.07	5.52	0.39	85.98
Woo (A,B,F)	56.28	91.60	41.20	95.25	6.70	0.48	88.26
Woo (A,F)	11.48	99.54	72.41	91.49	25.10	0.89	91.21
Woo (A,B)	82.51	73.20	24.35	97.56	3.08	0.24	74.08
Combs (A,H,C)	50.27	94.22	47.67	94.77	8.71	0.52	90.07
Warsof (A,F)^a^	32.24	98.06	63.44	93.26	16.59	0.69	91.83
Warsof (A,B)^b^	61.75	88.29	35.53	95.67	5.27	0.43	85.77
Vintzileos (A,B)	27.87	97.60	54.84	92.83	11.61	0.74	91.00
Shepard (A,B)^b^	20.22	98.97	67.27	92.22	19.66	0.81	91.52
Jordaan (A,B)^b^	36.61	96.80	54.47	93.59	11.44	0.65	91.10
Jordaan (A,H)	27.32	98.23	61.73	92.82	15.42	0.74	91.52
Jordaan (A,B,H)	46.45	95.77	53.46	94.48	10.98	0.56	91.10
Shinozuka (A,B,F)	36.61	97.71	62.62	93.65	16.02	0.65	91.93
Hsieh (A,B,F)	50.27	95.03	51.40	94.81	10.11	0.52	90.79
Ott (A,H,F)^b^	45.36	95.94	53.90	94.38	11.18	0.57	91.15
New staged models	81.97	98.00	81.08	98.11	40.98	0.18	96.48

SGA: small for gestational age; New staged models: VM model for 28–30, 31–33 and 34–36 weeks, MLR model for 37–39 weeks, FPR model for 40–42 weeks. A: abdominal circumference (AC); B: biparietal diameter (BPD); H: head circumference (HC); F: femur length (FL) expressed in cm; ^a^FL expressed in mm; ^b^EFW expressed in kg; PPV, positive predictive value; NPV, negative predictive value; +LR, positive likelihood ratio; −LR, negative likelihood ratio; Overall accuracy was calculated as (true positive + true negative)/total cases.

**Table 5:** Accuracy of staged birth weight prediction models for detection of LGA at birth.

Model	Sensitivity (%)	Specificity (%)	PPV (%)	NPV (%)	+LR	−NLR	Overall accuracy (%)
Hadlock (A,B,H,F)	33.09	96.74	62.76	89.71	10.16	0.69	87.69
Hadlock (A,H,F)	29.09	97.53	66.12	89.24	11.76	0.73	87.79
Hadlock (A,B,F)	35.64	96.02	59.76	90.00	8.95	0.67	87.43
Hadlock (A,H)	37.45	96.20	62.05	90.27	9.86	0.65	87.84
Hadlock (A,B)	51.64	93.12	55.47	92.07	7.51	0.52	87.22
Hadlock (A,F)	32.36	96.74	62.24	89.61	9.94	0.70	87.58
Intergrowth 21^st^ study (A,H)	22.91	98.37	70.00	88.50	14.07	0.78	87.64
Woo (A,B,F)	20.36	98.55	70.00	88.18	14.07	0.81	87.43
Woo (A,F)	90.55	62.85	28.77	97.57	2.44	0.15	66.79
Woo (A,B)	8.36	99.82	88.46	86.79	46.22	0.92	86.81
Combs (A,H,C)	18.91	98.43	66.67	87.98	12.06	0.82	87.12
Warsof (A,F)^a^	45.45	93.61	54.11	91.19	7.11	0.58	86.76
Warsof (A,B)^b^	23.64	97.95	65.66	88.55	11.53	0.78	87.38
Vintzileos (A,B)	70.18	83.45	39.88	94.34	4.00	0.36	80.70
Shepard (A,B)^b^	77.45	77.93	36.79	95.42	3.51	0.29	77.86
Jordaan (A,B)^b^	46.55	94.45	58.18	91.42	8.39	0.57	87.64
Jordaan (A,H)	42.91	93.24	51.30	90.78	6.35	0.61	86.08
Jordaan (A,B,H)	45.82	94.21	56.76	91.29	7.91	0.58	87.33
Shinozuka (A,B,F)	31.27	83.41	23.82	87.98	1.89	0.82	76.00
Hsieh (A,B,F)	43.27	94.63	57.21	90.96	8.06	0.60	87.33
Ott (A,H,F)^b^	30.18	97.53	66.94	89.39	12.21	0.72	87.95
New staged models	74.91	95.96	75.46	95.84	18.54	0.26	92.96

LGA: large for gestational age; New staged models: VM model for 28–30, 31–33 and 34–36 weeks, MLR model for 37–39 weeks, FPR model for 40–42 weeks. A: abdominal circumference (AC); B: biparietal diameter (BPD); H: head circumference (HC); F: femur length (FL) expressed in cm; ^a^FL expressed in mm; ^b^EFW expressed in kg; PPV, positive predictive value; NPV, negative predictive value; +LR, positive likelihood ratio; −LR, negative likelihood ratio; Overall accuracy was calculated as (true positive + true negative)/total cases.

**Table 6:** Accuracy of staged birth weight prediction models for detection of macrosomia at birth.

Model	Sensitivity (%)	Specificity (%)	PPV (%)	NPV (%)	+LR	−NLR	Overall accuracy (%)
Hadlock (A,B,H,F)	21.90	99.23	62.16	95.68	28.60	0.79	95.03
Hadlock (A,H,F)	20.95	99.34	64.71	95.63	31.92	0.80	95.09
Hadlock (A,B,F)	27.62	99.18	65.91	95.98	33.66	0.73	95.29
Hadlock (A,H)	18.10	99.34	61.29	95.48	27.57	0.82	94.93
Hadlock (A,B)	36.19	98.52	58.46	96.41	24.50	0.65	95.14
Hadlock (A,F)	24.76	99.07	60.47	95.82	26.63	0.76	95.03
Intergrowth 21^st^ study (A,H)	7.62	99.62	53.33	94.94	19.90	0.93	94.62
Woo (A,B,F)	18.10	99.56	70.37	95.49	41.35	0.82	95.14
Woo (A,F)	75.24	82.60	19.90	98.31	4.33	0.30	82.20
Woo (A,B)	2.86	99.95	75.00	94.71	52.23	0.97	94.67
Combs (A,H,C)	7.62	99.73	61.54	94.95	27.86	0.93	94.72
Warsof (A,F)^a^	35.24	98.03	50.68	96.34	17.89	0.66	94.62
Warsof (A,B)^b^	19.05	99.45	66.67	95.53	34.82	0.81	95.09
Vintzileos (A,B)	64.76	92.18	32.23	97.85	8.28	0.38	90.69
Shepard (A,B)^b^	65.71	91.03	29.61	97.88	7.32	0.38	89.65
Jordaan (A,B)^b^	31.43	98.91	62.26	96.17	28.73	0.69	95.24
Jordaan (A,H)	24.76	98.74	53.06	95.81	19.68	0.76	94.72
Jordaan (A,B,H)	36.19	98.30	55.07	96.41	21.34	0.65	94.93
Shinozuka (A,B,F)	19.05	99.45	66.67	95.53	34.82	0.81	95.09
Hsieh (A,B,F)	34.29	98.63	59.02	96.31	25.07	0.67	95.14
Ott (A,H,F)^b^	20.00	99.40	65.63	95.58	33.24	0.80	95.09
New staged models	78.10	93.87	42.27	98.68	12.75	0.23	93.02

New staged models: VM model for 28–30, 31–33 and 34–36 weeks, MLR model for 37–39 weeks, FPR model for 40–42 weeks. A: abdominal circumference (AC); B: biparietal diameter (BPD); H: head circumference (HC); F: femur length (FL) expressed in cm; ^a^FL expressed in mm; ^b^EFW expressed in kg; PPV, positive predictive value; NPV, negative predictive value; +LR, positive likelihood ratio; −LR, negative likelihood ratio; Overall accuracy was calculated as (true positive + true negative)/total cases.

### Comparison of new staged models and single models

New single models were also established by MLR, FPR and VM for all gestational ages based on the training group (Table S3). Table S4 showed the systematic errors and random errors of single models, which were higher than those of staged models for all newborns in the validation group. New single models had higher RMSE values and lower prediction rates within 1%, 5%, and 10% of birth weight than new staged models (Table S4). Comparisons of accuracy parameters of single models for prediction of SGA, LGA and macrosomia were displayed in Table S5. We observed that staged models had a better accuracy for prediction of SGA, LGA and macrosomia at birth.

## Discussion

Although many EFW models based on 2D ultrasound measurements have been formed, their accuracy was unsatisfied^22^. Developing the EFW formula requires as many pregnant women as possible who have a standardized ultrasound scan, and birth weight measurement^18^. To our knowledge, this study is the first report on birth weight prediction for different gestational age stages (from 28–30 to 40–42 weeks) in a large Chinese population including 19,310 newborns. We formed new staged birth weight prediction models, which were best established by VM, MLR and FPR model for first three gestational stages (28–30, 31–33 and 34–36 weeks), 37–39 weeks and 40–42 weeks, respectively.

For the first three gestational age stages, VM model showed the best prediction performance in the training group among three new models (VM, MLR, FPR) and previously published models (Fig. 1a–c). In 28–30 weeks, the systematic error of Hadlock (AB,F) model (0.23%) was lower than that of VM model (0.3%) and not significantly different from zero (*p* = 0.834), but the random error increased 7.85% (Fig. 1a). In 31–33 weeks, the systematic error of Hadlock (A,H,F) model (0.17%) increased 1.13 times compared to VM model (0.08%) (Fig. 1b). In 34–36 weeks, Hadlock (A,B,H,F) model had a second lower systematic error (−0.06%) which was the double of VM model (0.03%) (Fig. 1c). Furthermore, considering the comparisons with the previously published models in the validation group (Fig. 2a–c), it suggests that the VM model could provide more accurate prediction of birth weight for the first three stages.

For the last two gestational age stages, the systematic errors for previously published models were great higher than new staged models in the training group, and significantly different from zero with *p*-value all <0.05 (Fig. 1d,e). In 37–39 weeks, MLR model had the lowest systematic error (0.09%) and random error (7.23%) compared with FPR model (0.3%, 7.73%) and VM model (0.5%, 7.84%) (Fig. 1d). In 40–42 weeks, at least a 44.83% in the systematic error and a 9.03% in random error of FPR model were observed compared to MLR model and VM model (Fig. 1e). Meanwhile, according to the comparisons of prediction models in the validation group (Fig. 2d,e), the MLR and FPR model were considered as the best prediction models in the 37–39 and 40–42 weeks, respectively. The lowest aggregate systematic error and random error were also found in the new staged models (Table 3).

What’s more, it is acceptable if the prediction rate within 10% of birth weight was more than 80%^16^. In our study, the prediction rate within 10% of birth weight in new staged models was 85.1%, which was higher than previously published models. Furthermore, new staged models presented the better accuracy (sensitivity, specificity, PPV, NPV, +LR, −LR and overall accuracy) than previously published models for detection of SGA, LGA and macrosomia at birth. To further illustrate the accuracy of staged models, single models were developed using the same methods. Our results showed that single models presented the higher systematic errors, random errors, RMSE values, and the lower prediction rates within 1%, 5% and 10% of birth weight than staged models. The similar results of accuracy for single models and staged models for detection of SGA, LGA and macrosomia at birth were observed. It suggests that staged models had better performance than single models due to the varying growth velocity in different gestational age stages. Thus, we think that new staged models could improve the accuracy of birth weight estimation.

Dudley^23^ compared 11 EFW formulas and concluded that there was no preferred model for estimation of fetal weight due to population differences, maternal factors and measurement methods. To avoid the significant differences for fetal weight with race^14^, some studies reported the EFW models using ultrasound measurements in a Chinese population. Liao *et al*.^15^ established an EFW formula using 1,197 fetal biometrics who were delivered between 37 and 41 weeks. Yang *et al*.^16^ formed a new birth weight prediction model, in which 290 Hong Kong pregnant women who were delivered at 37–42 weeks were included. However, the prediction models were established using 2D and 3D ultrasound, and sample of two studies was not large and limited to the late third-trimester fetuses. Woo *et al*.^24^ developed an EFW formula with only 125 subjects whose detailed information was not included. It was reported that the prediction errors of Woo’s formula were higher than Hadlock’s formula^25^. Furthermore, our study showed that Woo’s model had higher systematic error, random error and RMSE value, and lower prediction rates within 1%, 5%, and 10% of birth weight than new staged models (Table 3).

Melamed *et al*.^26^ indicated that even the most precise models tend to the larger prediction errors. The potential sources of error are: first, observer differences. It is confirmed that ultrasound measurements, especially AC, are variable between operators, even with experience^27,28^. Second, because of different body composition, even the same circumference (AC) or length (FL) measurements may lead to different weight^29^. Third, fetal position is a factor that affects the measurement of fetal biometrics, which may be addressed by 3D ultrasound^15^. The use of 2D and 3D ultrasound measurements in the birth weight prediction will be needed for future study.

The subjects in this study were those who had both a delivery and an ultrasound 3 days prior to delivery such that it may cause selection bias in theory. However, with the universal use of sonographic technique in clinical practice, Chinese pregnant women receive regular ultrasound scan during pregnancy, especially prenatal ultrasonography which has become an essential part of prenatal diagnosis. Additionally, Cohen *et al*.^30^ found that more than 3 days of ultrasound-to-delivery intervals tended to affect the accuracy of EFW. Thus, the selection of population in this study is not bias in some extent. Nevertheless, it is remarkable that the application of new staged birth weight prediction models should be cautious in other population study, because many other countries (e.g., USA, UK) pregnant women are not routinely scanned in late pregnancy, but are selected for ultrasonography based on pre-pregnancy risk factors and obstetric complications^31,32^. Therefore, further studies should be undertaken to verify the accuracy of our new staged birth weight prediction models in other population studies, and explore the influence of population selection for different ultrasound-to-delivery intervals on birth weight prediction.

The strengths of this study are, first, it was a study with a large sample size including 1,9310 Chinese fetuses; second, the population was split into the training group and the validation group to better establish models and validate the prediction performance of models, respectively; third, we recruited the pregnant women who had an ultrasound scan within 3 days prior to delivery to avoid the prediction bias caused by wide ultrasound-to-delivery intervals^30^; fourth, we proposed the staged birth weight prediction models instead of single model, that is, developing the different prediction models for each gestational age stage. Some studies focused on the accuracy of fetal weight estimation for preterm or term fetuses^33,34^. For example, Hadlock’s^9^ and Warsof’s^11^ formulas usually underestimate preterm fetal weight, and Shepard’s^10^ formula was likely to overestimate fetal weight at term. This study showed the direct evidence that new staged prediction models could more accurately estimate birth weight.

There are several limits in our study. First, there are several types of ultrasound machines in the study, and the difference of ultrasound machines calibration may cause the slightly impact on the variability of the measurements^35^. Second, due to the smaller sample size for 28–30 weeks compared to others’, the accuracy of the fetal weight estimation may be affected before 30 weeks.

In conclusion, compared to the previously published models, new staged prediction models presented the higher accuracy in a Chinese population during 28–42 weeks. It suggests that new staged models could be more accurate than single formula on the birth weight prediction for given gestational age stage.

## Materials and Methods

### Study population

This was a retrospective cross-sectional study of all women who had an ultrasound examination within 3 days prior to delivery. The study subjects were from two sites: Wuhan Women and Children Medical Care Center (between May 2012 and June 2015), and Shenzhen Luohu Maternity and Children Health Care Hospital (between January 2011 and December 2015). The data was from healthcare information system of two hospitals. Inclusion and exclusion criteria were used: (1) singleton pregnancy, (2) delivery between 28–42 weeks’ gestation, (3) live birth without any congenital malformation, (4) complete measurements (HC, BPD, AC and FL). Gestational age was determined using the self-reported last menstrual period (LMP) if it agreed with the ultrasound estimation within 7 days; otherwise, the ultrasound estimation based on crown-lump length was used^36,37^.

All participants signed inform consents prior to engaging in any study activities. This study was approved by the ethics committee of Tongji Medical College, Huazhong University of Science and Technology, and Wuhan Women and Children Medical Care Center. All the research procedures were performed in accordance with relevant guidelines and regulations.

### Ultrasound measurements

The 2D ultrasound measurements for all participates included the following biometrics: BPD, HC, AC and FL, which were obtained from the ultrasound images and uploaded electronically to the data management system. The three types of ultrasound machines, an ALOKA SSD-5500SV (Tokyo, Japan), a Philips iu22 or HD15 (Bothell, WA, USA), and a GE Voluson E10 or E8 (Zipf, Austria), were used at two sites. Fetal biometrics consisting of BPD, HC, AC and FL were measured in millimeters during the ultrasound examination. BPD was measured from the outer border of the proximal parietal bone to the inner border of the distal parietal bone (“outer to inner”) at the widest part of the skull. HC was obtained by placing the calipers on the outer border of the skull and using the ellipse facility to follow the outer perimeter of the skull to calculate HC. AC measurements were taken at the outer surface of the skin line, using the ellipse facility. For FL, the calipers were placed at the ends of the ossified diaphysis without including the distal femoral epiphysis if it was visible. Birth weight was measured within 1 hour after birth by experienced obstetric nurses using standardized procedures.

To control the quality of fetal ultrasound measurements, ultrasound examinations were performed by experienced and certified sonographers with subspecialty training in ultrasound imaging according to a standard protocol. All the sonographers had their scan evaluated for quality control at the early period of the study.

### Statistical analysis

The participates were divided into two groups through employing random sampling method: training group and validation group, which were used to establish birth weight prediction models and validate the accuracy of models, respectively. The Student t test and Pearson Chi-square test was used to examine the clinical and demographic characteristics of two groups, including maternal age, maternal weight, maternal height, parity, gestational age, ultrasound-to-delivery intervals, newborn’s gender, and birth weight.

It was reported that the growth velocity of Chinese fetal weight showed an inversed “V” shape, and peaked at 34 weeks; furthermore, a significant difference in fetal weight was found at 28, 30, 32 and 38 weeks compared to the published Caucasian data^38^. To determine growth velocity, the “interval method” proposed by Guihard-Costa^39,40^ was used, that is, length of pregnancy was divided into 3-week intervals. Therefore, due to the effect of growth velocity on body weight, we divided gestational age into five stages, 3-week intervals per stage, that is, 28–30, 31–33, 34–36, 37–39 and 40–42 weeks. The multiple linear regression (MLR), fractional polynomial regression (FPR)^41,42^ and volume-based model (VM)^12,43^ were used to establish new birth weight prediction models for different gestational age stages. In the regression model, we also considered the interactions among BPD, HC, AC and FL. In volume-based model, fetal body weight was calculated by the sum of weight of fetal trunk and head. Based on physical and geometric theory, the volume of trunk was expressed as FL × AC^2^, thus, the weight of trunk was equal to be proportional to FL × AC^2^. Similarly, the weight of head was proportional to the volume of head, which was modeled as HC^3^, BPD^3^, BPD × HC^2^ and BPD^2^ × HC. The models, including the variables, the coefficients and the fractional polynomial powers (only for FPR), were elicited by the backward elimination algorithm.

We used two ways to evaluate the accuracy of new staged birth weight prediction models and 21 previously published EFW formulas^8–13,18,24,44–48^: (1) comparing systematic error, random error, root-mean-square error (RMSE) and proportion of prediction within 1%, 5% and 10% of actual birth weight for all prediction models. Systematic error was calculated as the mean of percentage error (PE) which was defined as ${\rm{PE}}=[({\rm{EFW}}-{\rm{Birth}}\,{\rm{Weight}})/{\rm{Birth}}\,{\rm{Weight}}]\times {\rm{100}} \%$. Random error was evaluated by the standard deviation (SD) of the PE; (2) comparing the sensitivity, specificity, positive predictive value (PPV), negative predictive value (NPV), positive likelihood ratio (+LR), negative likelihood ratio (−LR) and overall accuracy for detection of SGA, LGA and macrosomia. At last, in order to validate the performance of new staged birth weight prediction models, we established single models for all gestational ages in the training group and compared their accuracy in the validation group.

All the statistical analyses were carried out in R statistical software version 3.4.1 and SAS Software version 9.4. The statistical significance was set at an α level of 0.05 with a two-sided test.

## Supplementary information

Supplementary Tables S1–S5

## Data Availability

The datasets generated during and/or analyzed during the current study are available from the corresponding author on reasonable request.
