# Supplementary material for: Birth weight prediction models for the different gestational age stages in a Chinese population
Source: Sci Rep. 2019 Jul 25;9:10834. doi: 10.1038/s41598-019-47056-0 (PMC6658529; doi:10.1038/s41598-019-47056-0)
Supplement: Supplementary file 1 — Supplementary Tables S1–S5 [file 41598_2019_47056_MOESM1_ESM.pdf]

# Birth weight prediction models for the different gestational age stages in a Chinese population

Chunhui Li[1], Yang Peng[1], Bin Zhang[2], Weiying Ji[2], Li Li[3], Jianhua Gong[3], Wei Xia[1], Yuanyuan Li[1], Shuna Jin[1], Ranran Song[4], Youjie Wang[4*], Shunqing Xu[1*]

[1] Key Laboratory of Environment and Health (HUST), Ministry of Education & Ministry of Environmental Protection, School of Public Health, Tongji Medical College, Huazhong University of Science and Technology, Wuhan, Hubei, China

[2] Wuhan Medical and Health Center for Women and Children, Wuhan, Hubei, China

[3] Shenzhen Luohu Maternity and Children Health Care Hospital, Shenzhen, Guangdong, China

[4] Department of Maternal and Child Health, School of Public Health, Tongji Medical College, Huazhong University of Science and Technology, Wuhan, Hubei, China

Correspondence should be addressed to S.X. (email: xust@hust.edu.cn), Y.W. (email: wangyoujie@mails.tjmu.edu.cn)

**Table S1.** Comparisons of demographic characteristics between the training group and the validation group.

| Characteristics | Training Group<br>N (%) or Median (IQR) | Validation Group<br>N (%) or Median (IQR) | *p*-value |
|---|---|---|---|
| Maternal Characteristics | | | |
| Age | 28 (4) | 28 (5) | 0.359 |
| Weight[a] | 54 (10) | 54 (10) | 0.437 |
| Height[b] | 160 (8) | 160 (8) | 0.946 |
| Parity[c] | 14030 (81.02) | 1529 (79.39) | 0.084 |
| Gestational age | 39 (2) | 39 (2) | 0.975 |
| Ultrasound-to-delivery intervals | 1 (1) | 1 (1) | 0.524 |
| Newborn Characteristics | | | |
| Gender | | | 0.647 |
| Male | 9317(53.62%) | 1047(54.16%) | |
| Birth weight | 3300 (560) | 3300 (560) | 0.607 |

Continuous variables are presented as median (IQR: interquartile range); categorical values are N (%); [a]: 2606 missing; [b]: 5242 missing; [c]: 68 missing.

**Table S2.** Common ultrasonic EFW models.

| Model | Population | EFW formulas |
|---|---|---|
| Hadlock (A,B,H,F) | Caucasian | $\log_{10}EFW=1.3596-0.00386\ AC\times FL+0.0064\times HC+0.00061\times BPD\times AC+0.0424\times AC+0.174\times FL$ |
| Hadlock (A,H,F) | | $\log_{10}EFW=1.326-0.00326\times AC\times FL+0.0107\times HC+0.0438\times AC+0.158\times FL$ |
| Hadlock (A,B,F) | | $\log_{10}EFW=1.335-0.0034\times AC\times FL+0.0316\times BPD+0.0457\times AC+0.1623\times FL$ |
| Hadlock (A,H) | | $\log_{10}EFW=1.182+0.0273\times HC+0.07057\times AC-0.00063\times AC^2-0.000218\times HC\times AC$ |
| Hadlock (A,B) | | $\log_{10}EFW=1.1134+0.05845\times AC-0.000604\times AC^2-0.007365\times BPD^2+0.000595\times BPD\times AC+0.1694\times BPD$ |
| Hadlock (A,F) | | $\log_{10}EFW=1.304+0.05281\times AC+0.1938\times FL-0.004\times FL\times AC$ |
| Intergrowth 21st study (A,H) | Racial mix (Chinese included) | $\log EFW=5.08482-54.06633\times(AC/100)^3-95.80076\times(AC/100)^3\times\log(AC/100)+3.13637\times(HC/100)$ |
| Woo (A,B,F) | Hong Kong Chinese | $\log_{10}EFW=1.14+0.16\times BPD+0.05\times AC-0.0028\times BPD\times AC+0.04\times FL-0.00049\times FL\times AC$ |
| Woo (A,F) | | $\log_{10}EFW=0.59+0.08\times AC+0.28\times FL-0.00716\times FL\times AC$ |
| Woo (A,B) | | $\log_{10}EFW=1.63+0.16\times BPD+0.00111\times AC^2-0.0000859\times BPD\times AC^2$ |
| Combs (A,H,C) | Racial mix (Asian included) | $EFW=0.23718\times HC^3+0.03312\times FL\times AC^2$ |
| Warsof (A,F)[a] | No details | $\log_e EFW=2.792+0.108\times FL+0.0036\times AC^2-0.0027\times FL\times AC$ |
| Warsof (A,B)[b] | | $\log_{10}EFW=-1.599+0.144\times BPD+0.032\times AC-0.000111\times AC\times BPD^2$ |
| Vintzileos (A,B) | No details | $\log_{10}EFW=1.879+0.084\times BPD+0.026\times AC$ |
| Shepard (A,B)[b] | No details | $\log_{10}EFW=-1.7492+0.166\times BPD+0.046\times AC-0.002546\times AC\times BPD$ |
| Jordaan (A,B)[b] | White | $\log_{10}EFW=-1.1683+0.0377\times AC+0.095\times BPD-0.0015\times AC\times BPD$ |
| Jordaan (A,H) | | $\log_{10}EFW=0.9119+0.0488\times HC+0.0824\times AC-0.001599\times AC\times HC$ |
| Jordaan (A,B,H) | | $\log_{10}EFW=2.3231+0.02904\times AC+0.0079\times HC-0.0058\times BPD$ |
| Shinozuka (A,B,F) | Japanese | $EFW=1.6230\times BPD^3+0.23966\times FL\times AC^2$ |
| Hsieh (A,B,F) | | $\log_{10}EFW=2.7193+0.0094962\times AC\times BPD-0.1432\times FL-0.00076742\times AC\times BPD^2+0.001745\times FL\times BPD^2$ |
| Ott (A,H,F)[b] | No details | $\log_{10}EFW=-2.0661+0.04355\times HC+0.05394\times AC-0.0008582\times AC\times HC+1.2594\times FL/AC$ |

EFW: estimated fetal weight expressed in g; A: abdominal circumference (AC); B: biparietal diameter (BPD); H: head circumference (HC); F: femur length (FL) expressed in cm; [a]: FL expressed in mm; [b]: EFW expressed in kg.

**Table S3.** New single birth weight prediction models based on the training group for all gestational ages.

| Model | Birth weight prediction models |
| --- | --- |
| MLR | $\log_{10}EFW= 0.77535+0.06332\times AC+0.28654\times FL+0.00062856\times BPD\times AC+0.0001623\times HC\times AC-0.00755\times AC\times FL$ |
| FPR | $\log_{10}EFW=2.474-0.4299\times(AC/100)^{3}-0.01161\times\ln(AC/100)\times(AC/100)^{3}-0.6788\times\ln((AC\times FL)/100)-0.7935\times\ln((AC\times FL)/100)^{2}+0.6249\times((FL\times HC)/100)^{2}-0.1176\times((FL\times HC)/100)^{3}+0.06333\times(BPD\times HC/100)-3.085\times(AC\times HC/1000)+0.2664\times((AC\times HC)/1000)^{3}$ |
| VM | $EFW=0.09081\times BPD\times HC^{2}+0.2905\times FL\times AC^{2}$ |

Weight is expressed in g; BPD, HC, AC and FL are expressed in cm; MLR: multiple linear regression; FPR: fractional polynomial regression; VM: volume-based model; EFW: estimated fetal weight; HC: head circumference; AC: abdominal circumference; BPD: biparietal diameter; FL: femur length.

**Table S4.** Accuracy of new single birth weight prediction model for all newborns in the validation group.

| Model | Systematic error (%) | Random error (%) | RMSE | Prediction within (%) | | |
|---|---|---|---|---|---|---|
| | | | | 1% | 5% | 10% |
| MLR | 0.62* | 8.11 | 269.08 | 10.76 | 48.94 | 79.31 |
| FPR | 0.44* | 8.02 | 267.30 | 10.35 | 48.47 | 79.67 |
| VM | 0.88* | 8.22 | 272.35 | 10.40 | 48.42 | 78.43 |

MLR: multiple linear regression; FPR: fractional polynomial regression; VM: volume-based model; RMSE: root mean square error; * indicates significantly different from zero ($p<0.001$).

**Table S5.** Accuracy of new single birth weight prediction models for detection of SGA, LGA and macrosomia at birth.

| Outcome | Model | Sensitivity (%) | Specificity (%) | PPV (%) | NPV (%) | +LR | -NLR | Overall accuracy (%) |
|---|---|---|---|---|---|---|---|---|
| SGA | | | | | | | | |
| | MLR | 62.84 | 88.69 | 36.74 | 95.80 | 5.55 | 0.42 | 86.24 |
| | FPR | 61.75 | 89.03 | 37.05 | 95.70 | 5.63 | 0.43 | 86.45 |
| | VM | 66.67 | 98.51 | 82.43 | 96.58 | 44.87 | 0.34 | 95.50 |
| LGA | | | | | | | | |
| | MLR | 56.00 | 95.36 | 66.67 | 92.89 | 12.09 | 0.46 | 89.76 |
| | FPR | 57.82 | 95.42 | 67.66 | 93.17 | 12.61 | 0.44 | 90.07 |
| | VM | 57.82 | 95.11 | 66.25 | 93.15 | 11.83 | 0.44 | 89.81 |
| Macrosomia | | | | | | | | |
| | MLR | 72.38 | 98.58 | 74.51 | 98.42 | 50.88 | 0.28 | 97.15 |
| | FPR | 73.33 | 98.25 | 70.64 | 98.46 | 41.89 | 0.27 | 96.89 |
| | VM | 72.38 | 99.01 | 80.85 | 98.42 | 73.51 | 0.28 | 97.57 |

SGA: small for gestational age; LGA: large for gestational age; MLR: multiple linear regression; FPR: fractional polynomial regression; VM: volume-based model; PPV, positive predictive value; NPV, negative predictive value; +LR, positive likelihood ratio; -LR, negative likelihood ratio; Overall accuracy was calculated as (true positive + true negative)/total cases.
